# Laparoscopy versus open surgery for adnexal masses in pregnancy: a meta-analytic review

**DOI:** 10.1007/s00404-018-05039-y

**Published:** 2019-01-31

**Authors:** Piaopiao Ye, Na Zhao, Jing Shu, Heping Shen, Yanpeng Wang, Lifeng Chen, Xiaojian Yan

**Affiliations:** 1 Department of Gynecology, The First Affiliated Hospital of Wenzhou Medical University, Wenzhou, 325000 China; 2 Department of Gynecology, Zhejiang Provincial People’s Hospital, Hangzhou, 310014 China

**Keywords:** Laparoscopy, Open surgery, Adnexal masses, Pregnancy, Meta-analysis

## Abstract

**Purpose:**

The objective of this meta-analysis is to investigate and compare the pregnancy outcomes of laparoscopy and open surgery in the treatment of ovarian tumors during pregnancy.

**Methods:**

Search was conducted using MEDLINE, EMBASE, and Cochrane Databases from January 1990 to November 2018. A broad search strategy was used to identify studies comparing laparoscopy and open surgery in pregnancy. Inclusion criteria included comparative studies with the quantitative outcome data on gravida. Two authors independently reviewed and assessed for the quality of included studies according to the Newcastle–Ottawa Scale. Data were extracted for fetal loss, preterm delivery, duration of surgery, blood loss and length of hospital stay.

**Results:**

Nine retrospective trials were identified involving 985 patients. No statistical significance was found in fetal loss between laparoscopy and open surgery (*P* value = 0.334). The pooled estimate for preterm labor statistically significantly decreased for laparoscopy group (*P* value = 0.014). Reduced operative blood loss was found in laparoscopy group by 83.81 ml (*P* value = 0.015). Duration of operation may be longer in the laparoscopy group, but without statistical significance (*P* value = 0.346). Length of hospital stay was shorter in the laparoscopy group with reduction of 1.95 days (*P* value < 0.001).

**Conclusions:**

The available low-grade evidence suggests that laparoscopic surgery might be a feasible alternative for pregnant women with adnexal masses.

## Introduction

The incidence of adnexal mass in pregnancy is about 41 in 1500 pregnancies [1]. These tumors are mostly benign, corpus luteum cyst is the most common type, it will disappear in 90% cases by the second trimester of pregnancy, followed by serous cystadenoma and dermoid cyst, which is the most common pathologies found [2–5]. Only approximately 1 in 25,000 pregnancies were observed to be malignant ovarian tumors [6]. Surgery is deemed to be dangerous for both the mother and the fetus, especially in emergency situations [2, 7, 8], which may lead to a high incidence of maternal complications, fetal death and premature birth [9]. However, for pregnant women with acute pelvic pain or an adnexal mass greater than 6 cm in diameter, selective surgical excision is not disputed. As the pregnancy progresses, they may occur in torsion, rupture, or leakage of the cyst, which may cause damage to both the mother and the fetus [2, 10]. In all, ovarian tumors in pregnancy requiring surgical intervention vary from 0.0004 to 0.36% [11, 12]. The procedures include resection of the tumor, oophorectomy, or salpingo-oophorectomy and so on. And the best surgical approach for a pregnancy with adnexal masses remains controversial. Since the mid-1990s, laparoscopy has been widely used in non-pregnant women’s gynecologic diseases. Nowadays, a growing number of evidence shows that laparoscopy can be safely and effectively used during pregnancy, and provides several advantages, including reduced postoperative pain, analgesic use and hospitalization time [13–19]. At the same time, some surgeons have been hesitant to perform minimally invasive surgery on pregnant patients. Potential concerns associated with pregnancy laparoscopic surgery include limited surgical manipulations, perforation of gravid uterus and hypercarbia [20, 21]. Laparoscopic surgery for a pregnancy with adnexal mass has been limited to case reports and retrospective studies in the last decade [9, 22–25]. No prospective controlled studies have been reported yet. Therefore, a meta-analysis was conducted.

The main purpose was to investigate and compare the pregnancy outcomes of two methods in the treatment of ovarian tumors during pregnancy, including fetal loss rate, premature delivery rate, operative time, bleeding volume and hospital stay.

## Methods

### Search strategy and eligibility criteria

A literature search was performed by searching MEDLINE, EMBASE, and Cochrane Databases to obtain comparative studies assessing the safety and efficiency between laparoscopy and laparotomy in women undergoing surgery for adnexal mass during pregnancy. The following mesh search headings were used: (“laparoscopic” or “laparoscopy”) and (“abdominal” or “laparotomy”) and (“pregnancy” or “pregnant” or “gravida”) and (“comparative studies and adnexal mass, and surgery”). Searches were also performed under the terms “laparoscopic versus open” and “minimally invasive versus conventional”. There was no restriction by language, or “publication status applied”. The “related articles” function was used to broaden the search, and all abstracts, studies, and citations scanned were reviewed. The latest date for this search was November 30, 2018.

### Inclusion criteria

To enter our analysis, studies had the following criteria:

1. It was a comparative study between LA and OP with the quantitative outcome data on gravida.
2. Report on at least one of the outcome measures mentioned below.
3. If the same institution and/or authors reported more than one study, we enrolled the larger scale number studies or higher quality studies.
4. Study was published in English.

### Exclusion criteria

The following criteria were used to exclude studies from our analysis:

1. Studies in which the outcomes of interest (mentioned below) were not reported for the two techniques or it was impossible to extract these from the published results.
2. Studies that used variations on the standard laparoscopic technique, including hybrid procedures (laparoscope-assisted) and single trocar techniques.
3. There was considerable overlap between authors, centers, or patient cohorts evaluated in the published literature.

### Data extraction

Two reviewers independently extracted the following data from each study: first author, year of publication, study design, mean age, intraoperative data (operative time, blood loss) and postoperative data (hospital stay) and outcomes (pregnancy outcome). Inconsistencies between reviewers’ data were resolved through discussion until a consensus was reached. The quality of included studies was estimated according to the Newcastle–Ottawa Scale (http://www.ohri.ca/programs/clinical_epidemiology/oxford.asp).

### Statistical analysis

This meta-analysis was carried out using the Stata version 11. All *P* values were two-sided and a *P* value of less than 0.05 was deemed statistically significant. In the included studies, if continuous data described as medians and ranges, these data were analyzed by approach reported by Hozo et al. [26] to calculate mean and standard deviations (SD). Sometimes it is desirable to combine two reported subgroups into a single group, we use the formulae for combining groups according to Cochrane Handbook for Systematic Reviews of Interventions (2008) [27]. Continuous variables used weighted mean difference (WMD) and a 95% confidence interval (CI). The relative risk (RR) with a 95% confidence interval (CI) was calculated for dichotomous outcomes (fetal loss and preterm delivery). If one cell in the 2 × 2 table contained zero, a continuity correction was carried by adding 0.5 to each cell [28, 29]. A fixed effect mode or a random effects model mode was applied. Heterogeneity was evaluated by *x*^2^-test and *I*^2^. We considered heterogeneity to be present if the *I*^2^ statistic was > 50%, and a random effect model were adopted. However, if *I*^2^ statistic was < 50%, we used a fixed effect model. *P* < 0.05 was considered to be significant. Funnel plot was used to evaluate publication of bias. In addition, funnel plot asymmetry was assessed by the method of Egger’s linear regression test [30].

## Results

Finally, nine retrospective trials with a total of 985 patients were included into this meta-analysis [8, 15, 17, 22, 24, 31–34]. In the 985 patients, 549 had undergone laparoscopic surgery and 436 had undergone open surgery. All studies included were published in English. The baseline characteristics and quality assessment of all included studies are listed in Table 1. All the included studies mentioned the mean age, and seven studies described gestational age at surgery. In this meta-analysis, we found that there was no statistical difference in age and gestational age between two groups. The mean patient age ranged from 27.0 to 31.5 years. Gestational age at diagnosis was mostly in the second trimester. Fetal loss and preterm labor were reported in all nine studies. Eight studies reported on operation time and seven reported hospital stay, and four reported blood loss.

**Table 1:** The baseline characteristics and quality assessment of studies

References	Quality score	Design	No. of women LA versus OP	Age (years) LA versus OP	Gestational (weeks) LA versus OP	Outcomes
Ngu [8]	8^a^	R	21 versus 14	31.4 ± 4.3 versus 31.6 ± 7.0	15.1 ± 1.8 versus 15.4 ± 2.2	Fetal loss, preterm delivery, hospital stay, duration of operation, birth weight, lost blood. mass size
Koo [17]	8^a^	R	88 versus 174	30.1 ± 3.6 versus 29.4 ± 3.3	11.6 ± 3.1 versus 15.1 ± 4.5	Apgar score, fetal loss, preterm delivery, hospital stay, duration of operation, mass size, birth weight
Chang [34]	8^a^	R	12 versus 8	29.1 ± 4.8 versus 29.8 ± 5.8	NA	Fetal loss, preterm delivery, hospital stay, duration of operation, lost blood
Balthazar [15]	9^a^	R	50 versus 51	27.6 ± 5.5 versus 25.4 ± 5.7	17.6 ± 0.4 versus 17.5 ± 0.5	Preterm delivery, hospital stay, duration of operation, blood lost, birth weight
Lee [22]	6^a^	R	17 versus 17	30.0 ± 3.5 versus 28.5 ± 3.0	12.9 ± 2.2 versus 12.4 ± 3.5	Apgar score, fetal loss, preterm delivery, hospital stay, duration of operation, lost blood
James [33]	6^a^	R	7 versus 9	27.1 ± 3.7 versus 22.9 ± 5.3	15.0 ± 6.0 versus 13.0 ± 4.0	Fetal loss, preterm delivery, duration of operation, hospital stay
Oelsner [32]	7^a^	R	192 versus 197	28.9 ± 6.0VS28.3 ± 5.5	NA	Fetal loss, duration of operation, hospital stay, birthweight
Akira [31]	8^a^	R	17 versus 18	30.6 ± 4.9 versus 29.7 ± 5.1	13.9 ± 1.4 versus 14.1 ± 2.1	Fetal loss, preterm delivery, duration of operation
Soriano [24]	7^a^	R	39 versus 54	28.3 ± 3.4 versus 27.0 ± 2.9	8.3 ± 1.7 versus 14.7 ± 5.9	Fetal loss, preterm delivery, birth weight

*NA* not available

^a^Values are expressed as mean ± SD

### Fetal loss

**Fig. 1:**
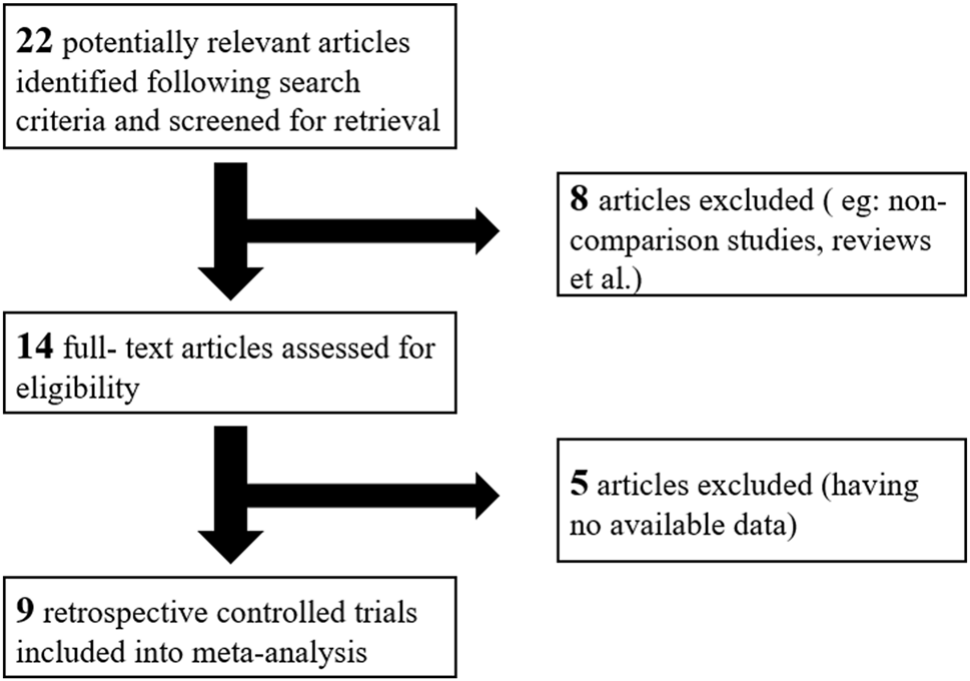
Flow chart demonstrating selection of studies for inclusion in the meta-analysis

**Fig. 2:**
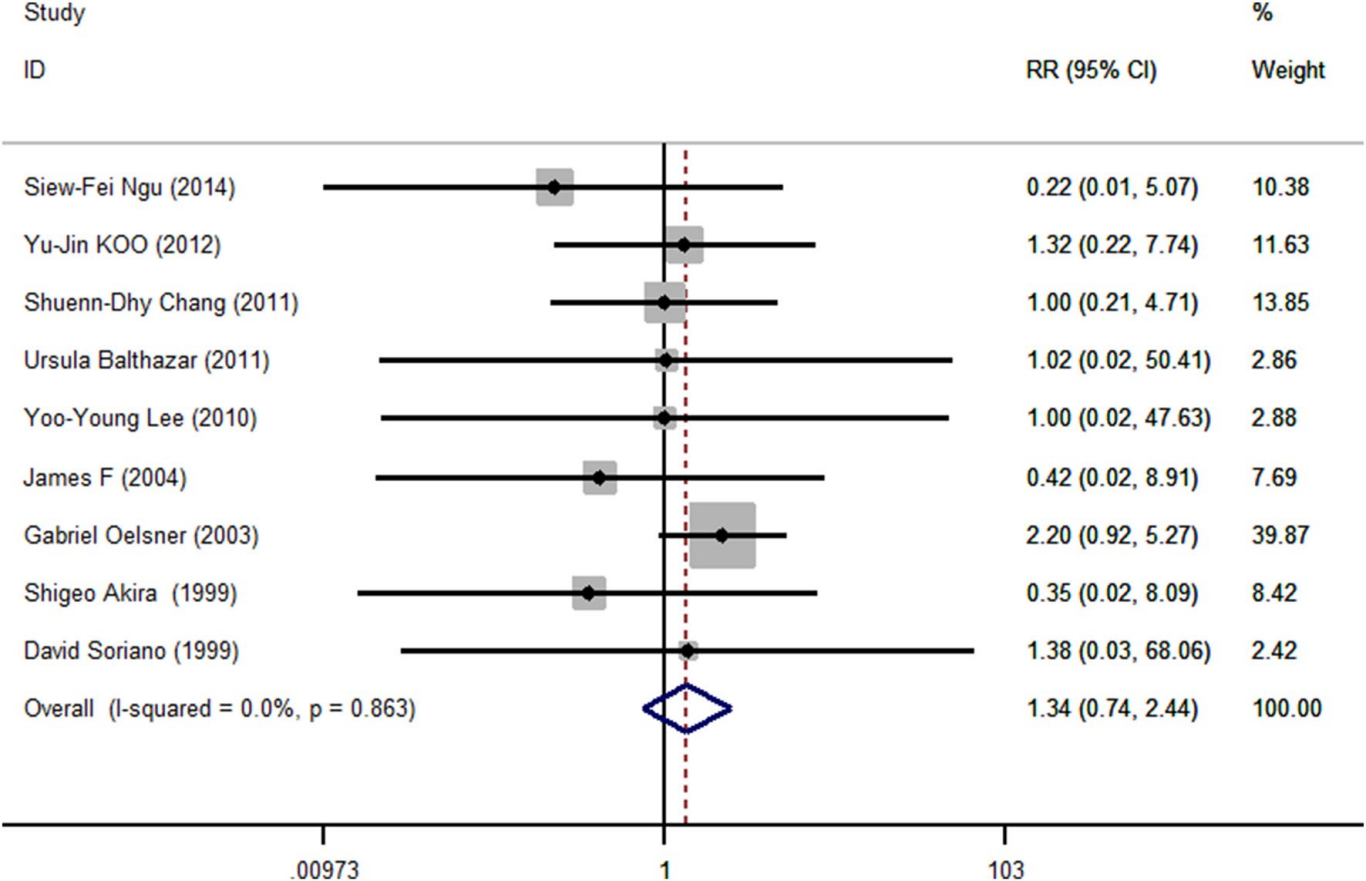
Meta-analysis of pregnancy outcomes fetal loss after laparoscopic (LA) versus open (OA) surgery. Relative risks are shown with 95% confidence intervals

**Fig. 3:**
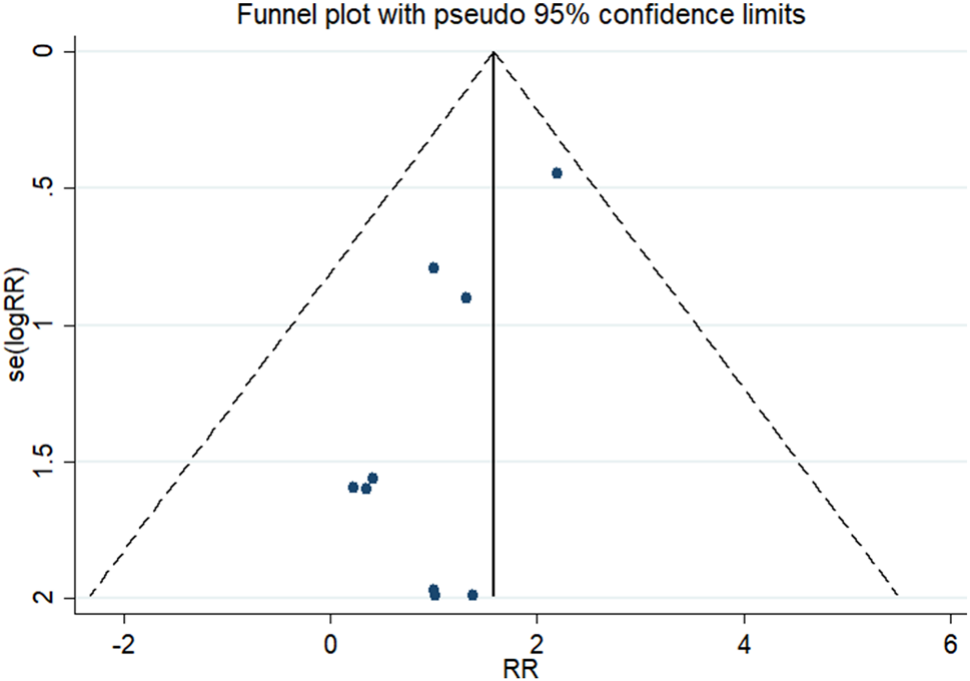
Contour-enhanced funnel plots for studies comparing fetal loss after laparoscopic versus open surgery

As shown in Fig. 1, the search strategy identified nine controlled trials that compared the results of laparoscopy to open surgery for adnexal mass during pregnancy. All nine studies reported fetal loss after surgery, which allowed quantitative pooled analysis. The RRs were homogeneous across studies *Q* = 3.93, *P* = 0.863, *I*^2^ = 0.0% < 50% with a pooled value (laparoscopy versus open surgery) of 1.36 (95% CI 0.73–2.55, *P* = 0.334). Forest plots displaying the results of the meta-analysis for fetal loss are shown in Table 2 and Fig. 2. This suggested that the odds of fetal loss in the laparoscopy were almost as same as the open surgery. Egger’s test suggested asymmetry of the funnel (*t* = − 3.60, *P* = 0.009). A contour-enhanced funnel plot was, therefore, created (Fig. 3). The funnel plot revealed an apparent asymmetry that prompted the presence of a potential publication bias, a language bias, inflated estimates by a flawed methodologic design in smaller studies, and/or a lack of publication of small trials with opposite results.

**Table 2:** Comparisons of fetal loss and preterm labor between laparoscopic and open surgery in pregnancy

References	Laparoscopic		Open		Relative risk
	Yes	No	Yes	No	
*Fetal loss*					
Ngu [8]	0	20	1	12	0.22 (0.01–5.08)
Koo [17]	2	86	3	171	1.32 (0.22, 7.75)
Chang [34]	3	9	2	6	1.00 (0.21, 4.71)
Balthazar [15]	0	50	0	51	1.02 (0.02, 50.41)
Lee [22]	0	17	0	17	1.00 (0.02, 47.63)
James [33]	0	7	1	8	0.42 (0.02, 8.91)
Oelsner [32]	15	177	7	190	2.20 (0.92, 5.27)
Akira [31]	0	17	1	17	0.35 (0.02, 8.09)
Soriano [24]	0	39	0	54	1.38 (0.03, 68.06)
Pooled relative risk					1.36 (0.73, 2.55)
*Preterm delivery*					
Ngu [8]	2	18	1	12	1.30 (0.13–12.92)
Koo [17]	2	86	33	141	0.12 (0.03, 0.49)
Chang [34]	2	10	1	7	1.33 (0.14, 12.37)
Balthazar [15]	3	47	3	48	1.02 (0.22, 4.82)
Lee [22]	1	16	1	16	1.00 (0.07, 14.72)
James [33]	1	6	3	4	0.33 (0.05, 2.48)
Oelsner [32]	5	187	6	191	0.86 (0.27, 2.76)
Akira [31]	0	17	0	18	1.06 (0.02, 50.43)
Soriano [24]	4	35	4	50	1.39 (0.37, 5.20)
Pooled relative risk					0.51 (0.30, 0.87)

### Preterm labor

**Fig. 4:**
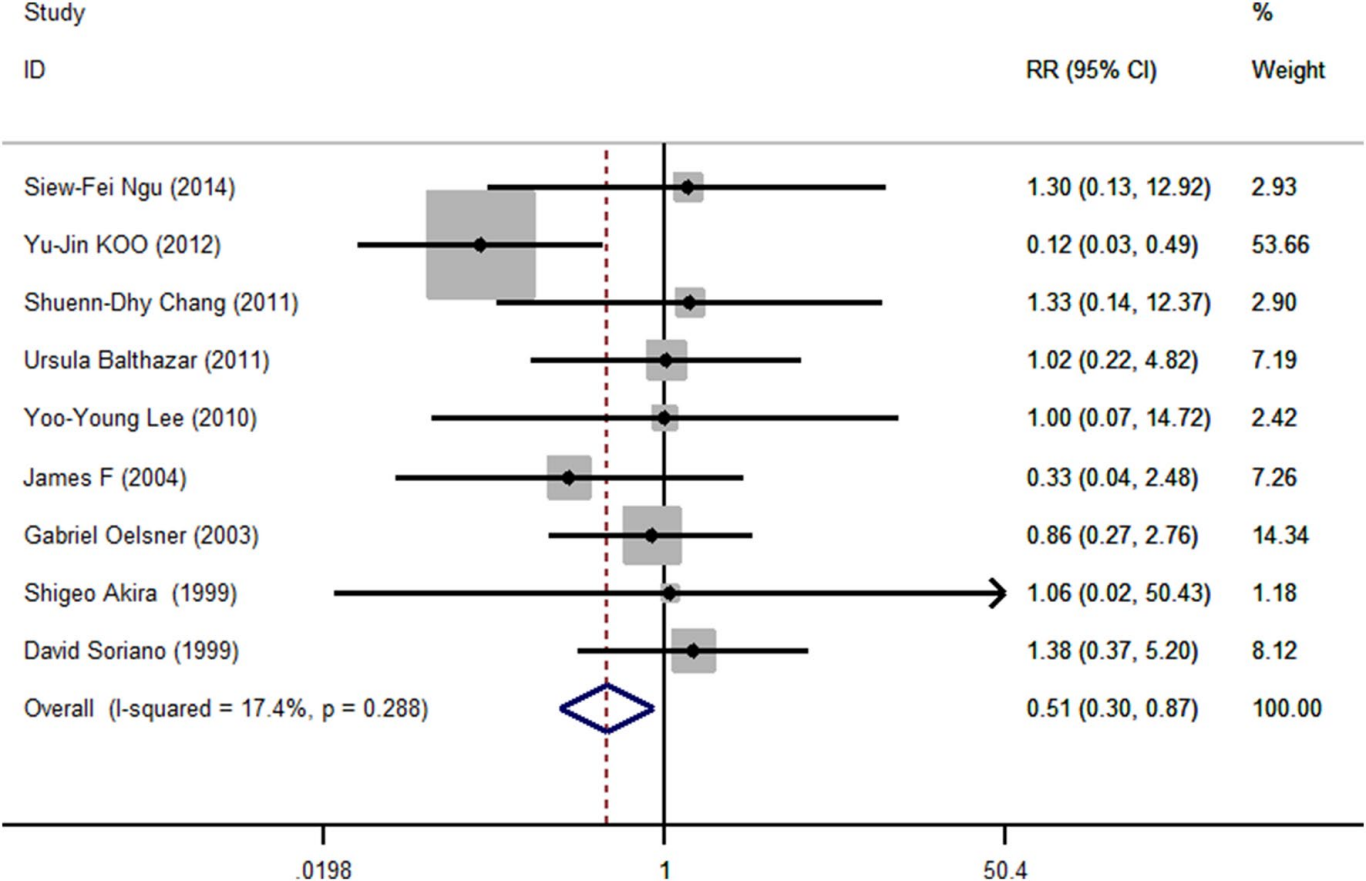
Meta-analysis of pregnancy outcomes preterm labor after laparoscopic (LA) versus open (OA) surgery. Relative risks are shown with 95% confidence intervals

**Fig. 5:**
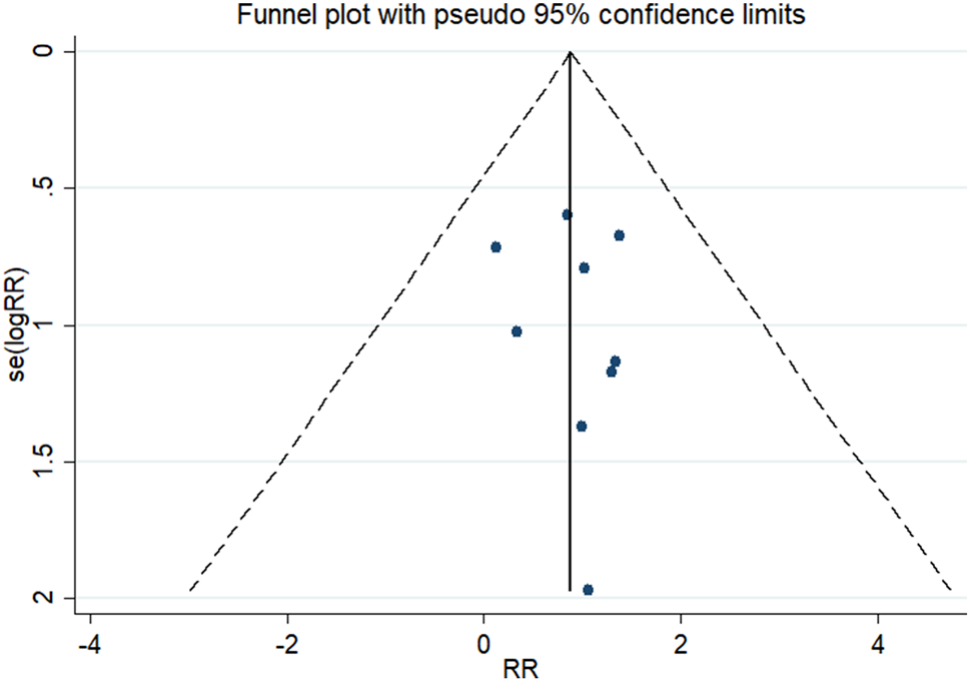
Contour-enhanced funnel plots for studies comparing preterm labor after laparoscopic versus open surgery

All nine studies reported preterm labor. The RRs were homogeneous *Q* = 9.69, *P* = 0.288, *I*^2^ = 17.4% < 50%. The pooled RR was 0.510 (95% CI 0.299–0.871, *P* = 0.014). (Table 2, Fig. 4), indicating that the odds of preterm labor was 51% lower in the laparoscopy than the open surgery group (*P* = 0.01); Egger’s test did not suggest publication bias (*t* = 0.37, *P* = 0.721) and this was supported by a symmetrical contour-enhanced funnel plot (Fig. 5).

### Operation time

**Fig. 6:**
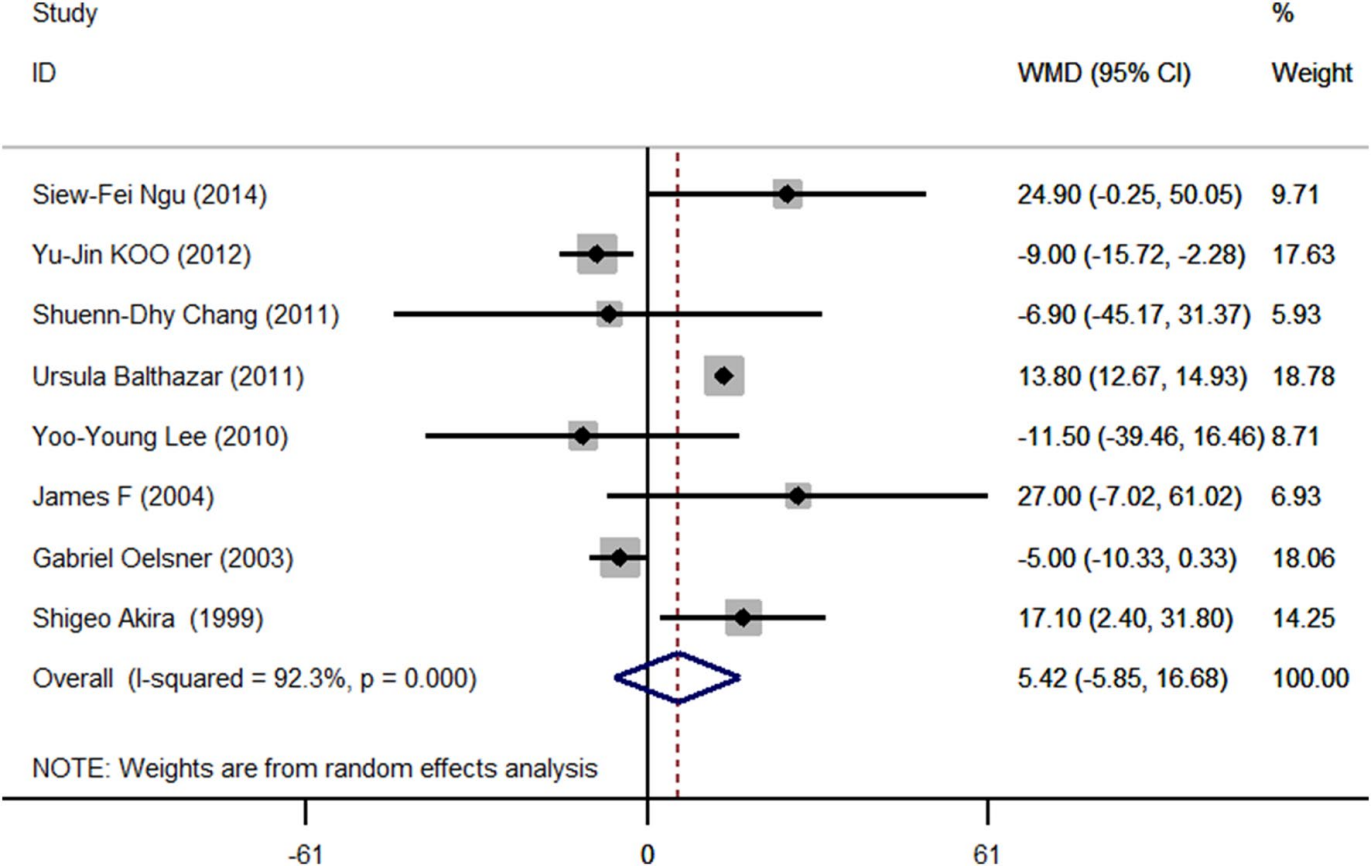
Meta-analysis of operation time in laparoscopic (LA) versus open (OA) surgery. Relative risks are shown with 95% confidence intervals

Duration of operation was also pooled across studies (Table 3). The duration of operation in the laparoscopy and open groups was compared in eight studies (*n* = 892). The data were heterogeneous *Q* = 91.47, *P* < 0.001, *I*^2^ = 92.3% > 50%, with an unstandardized mean difference of 5.42 (− 5.85 to 16.68), indicating that the duration of operation was not significant between LA group and OP group (*P* = 0.346) (Fig. 6). Egger’s test did not suggest publication bias (*P* = 0.249).

**Table 3:** Comparisons of duration of operation, blood loss and hospital stay between laparoscopic and open surgery in pregnancy

References	Operation time (min)		Blood lost (ml)		Birth weight (g)		Hospital stay (days)	
	LA	OP	LA	OP	LA	OP	LA	OP
Ngu [8]	92.5 ± 44.4	67.6 ± 29.3	67.4 ± 55.8	153.6 ± 181.0	3188.8 ± 343.5	3163.6 ± 445.6	2.8 ± 1.0	3.8 ± 1.1
Koo [17]	60.7 ± 27.1	69.7 ± 24.4	NA	NA	3174.7 ± 539.8	3197.2 ± 554.2	4.7 ± 1.7	6.6 ± 1.3
Chang [34]	87.9 ± 39.9	94.8 ± 44.6	58.8 ± 32.1	53.8 ± 60.9	NA	NA	2.9 ± 1.1	7.6 ± 7.9
Balthazar [15]	76.6 ± 3.1	62.8 ± 2.7	17.5 ± 1.6	101.5 ± 8.3	NA	NA	0.7 ± 0.1	2.8 ± 0.2
Lee [22]	106.3 ± 31.3	117.8 ± 49.8	152.5 ± 97.5	275.0 ± 125.0	NA	NA	3.5 ± 1.0	6.5 ± 1.0
James [33]	116 ± 34	89 ± 35	Minimal	117 ± 75	NA	NA	1.0 ± 0.0	4.4 ± 1.1
Oelsner [32]	± 25	64 ± 28.6	NA	NA	NA	NA	2.7 ± 2.1	4.3 ± 2.0
Akira [31]	75.4 ± 18.5	58.2 ± 25.5	NA	NA	NA	NA	NA	NA
Soriano [24]	NA	NA	NA	NA	NA	NA	NA	NA

Values are expressed as mean ± SD

*NA* not available

### Blood loss

**Fig. 7:**
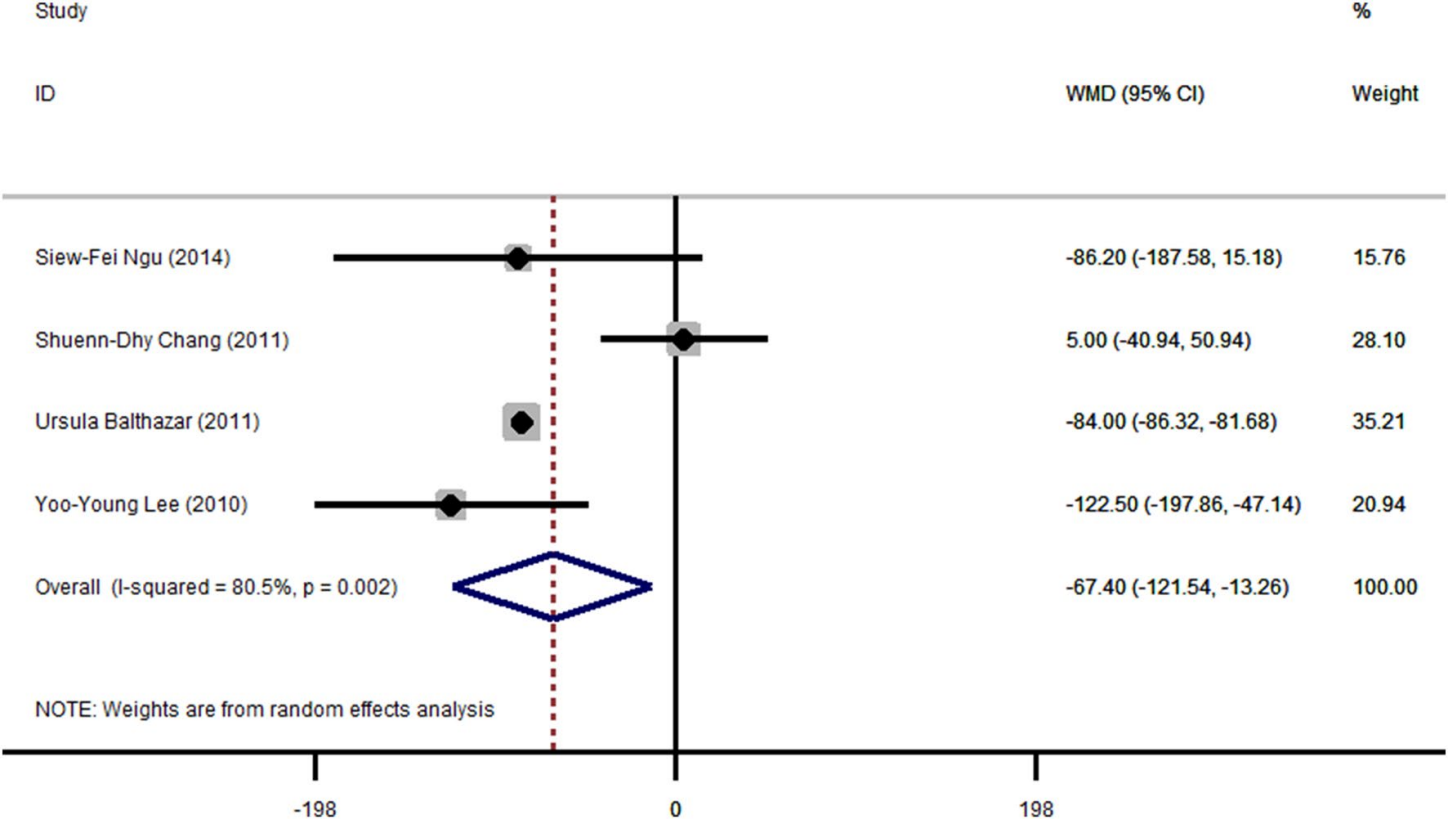
Meta-analysis of blood loss in laparoscopic (LA) versus open (OA) surgery. Relative risks are shown with 95% confidence intervals

Among four studies reported on blood loss during operation, one was discarded for no datum, three were meta-analyzed. It was found to be significantly lower in the laparoscopy group versus the open group by 83.81 ml (95% CI − 121.54 ~ − 13.26, *P* = 0.015) (Fig. 7) with significant heterogeneity *Q* = 91.47, *P* < 0.001, *I*^2^ = 92.3% > 50%. Egger’s test did not suggest publication bias (*P* = 0.606).

### Hospital stay

**Fig. 8:**
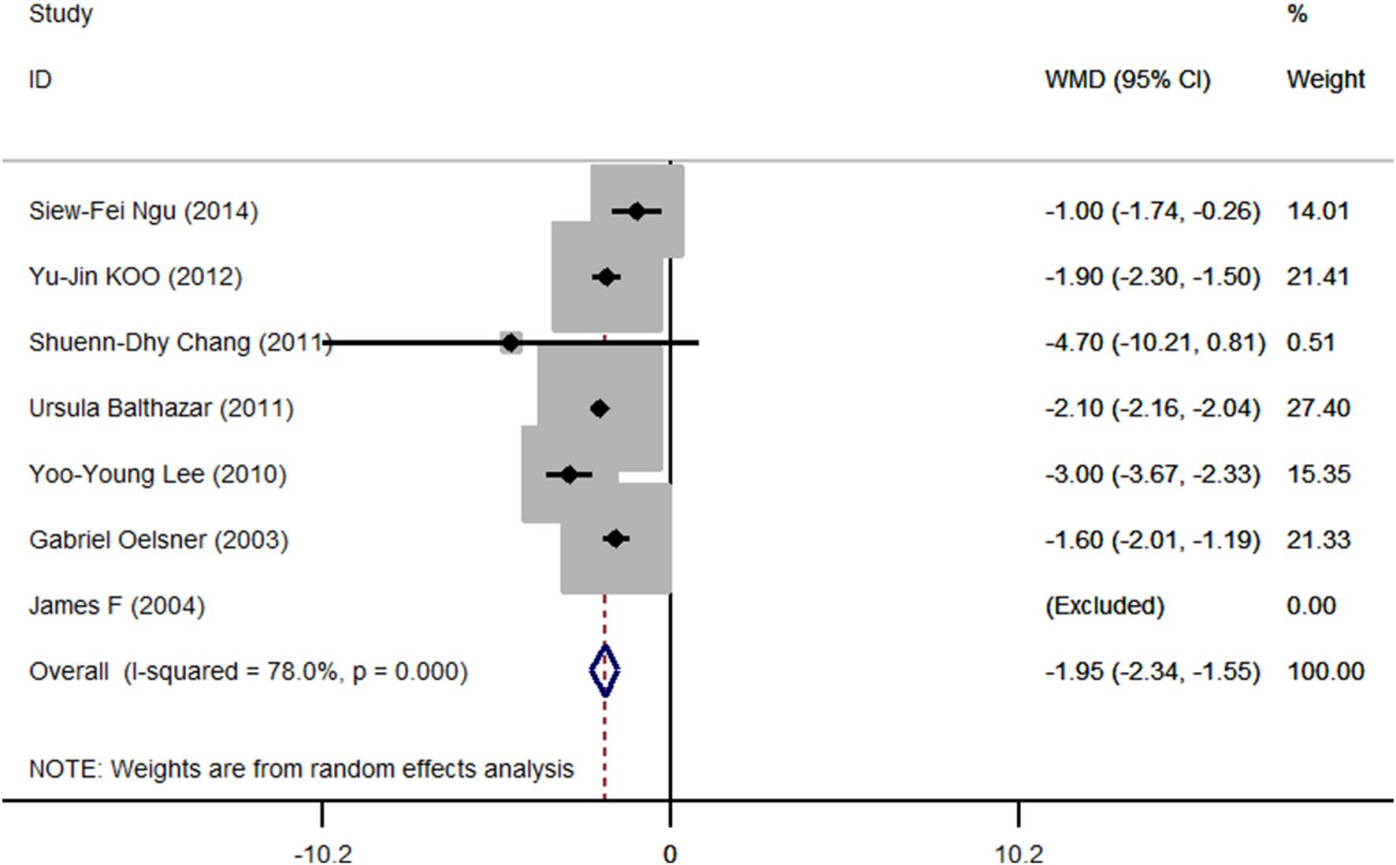
Meta-analysis of hospital stay in laparoscopic (LA) versus open (OA) surgery. Relative risks are shown with 95% confidence intervals

There were six studies that reported hospital stay while one was excluded for being unable to obtain valid SD. The length of hospital stay was significantly shorter in the laparoscopy group by almost 2 days (95% CI − 2.34 to 1.55, *P* < 0.001) (Fig. 8). Egger’s test did not suggest publication bias (*P* = 0.682).

## Discussion

Our systematic reviews and meta-analysis investigated all controlled clinical trials according to the inclusion criteria. The search strategy employed in the present meta-analysis was broad. Those derived from searching proceedings databases were not specifically excluded. According to the quality evaluation of Newcastle–Ottawa Scale results (NOS) for meta-analysis of non-randomized studies, the quality of most of the studies was considered to be high. The results of this meta-analysis suggest that laparoscopic surgery in pregnancy results in almost 51% lower risk of preterm labor, shorter hospital stay and lower blood loss compared with open surgery. No significant difference in fetal loss and operation time was observed between the two groups.

To date, a considerable number of studies demonstrate that laparoscopic surgery during pregnancy has been performed successfully for many conditions, such as cholecystectomy and appendectomy [18, 19, 35, 36], which have an advantage of good maternal outcomes, such as earlier ambulation, less pain after surgery and shorter hospital stay than open surgery [37]. At the same time, previous controlled studies have shown that it is not associated with higher rates of abortion and preterm deliveries in comparison with laparotomy [33, 34]. A meta-analysis has been published to review the effects of laparoscopic and open appendectomy in pregnancy [29]. However, previous randomized studies of laparoscopy versus open surgery in pregnant patients with adnexal mass are limited.

The risk of fetal loss has become the top priority in many studies of the relative safety of laparoscopy in pregnancy [38, 39]. The main consideration is laparoscopy requires carbon dioxide pneumoperitoneum [40]. Increased intra-abdominal pressure can lead to reduced uterine blood flow and maternal venous return, resulting in the fetal intrauterine hypoxia [41, 42]. Another factor associated with pneumoperitoneum is that carbon dioxide can be absorbed across the peritoneum, causing fetal acidosis [43]. However, Curet MJ hold no substantial adverse effect on the fetus when the maximum pressure of the pneumoperitoneal is less than 12 mmHg and the duration is less than 30 min [40]. To avoid this risk, the gasless laparoscopic technique was, therefore, recommended for pregnancy surgery [20, 31, 44, 45]. Another concern for the application of laparoscopic surgery during pregnancy is the risk of injury to the enlarged uterus [46, 47]. In the study of Balthazar [15], initial port was placed through an open method (80%) or a left upper quadrant entry (11%), thereby reducing the potential risk of penetrating injury to the gravid uterus. In all, although there is no statistical significance, the present results suggest that the risk of fetal loss may be increased in those undergoing laparoscopic surgery compared with open surgery. It is likely that this analysis did not have enough statistical capabilities to detect a significant difference, because a sample size of 985 would be required in each group to detect a RR of 1.36.

The risk of preterm labor after laparoscopy compared with open surgery has been discussed in many reports of the relative safety of laparoscopy in pregnancy [32, 48, 49]. The relative risk (RR) of the preterm labor between laparoscopy versus open surgery in this study was estimated as carried out by Wilasrusmee et al. [29], because the number of fetal loss is not excluded in data processing; it is difficult to reach a conclusion that laparoscopy has an advantage in preterm labor though the result indicates that the odds of preterm labor was 51% lower in the laparoscopy than the open surgery group (*P* = 0.014). In this study the increase of operating time in laparoscopic surgery is not statistically significant, probably due to the influence of the learning curve. Meanwhile, similar to the findings in non-gravid patients, laparoscopy was associated with improved short-term operative outcomes including decreased blood loss and shorter hospital stay. The results showed that the amount of blood loss (83.81 ml, *P* = 0.00) in the laparoscopy group was significantly reduced, which may attribute to the better visualization of deep vascular structures, and possibly more precise and accurate surgery. The length of hospital stay was approximately 2 days shorter in laparoscopy than that of open surgery (*P* < 0.000). However, these results should be interpreted with caution as the total number of patients is small and significant heterogeneity.

Meta-analytical research has several limitations that must be taken into account when its results are considered. One major potential limitation here is that all studies included in the review were observational, and summary data published within each study were included in the analysis. While not all cases in the studies were adnexal mass, some were found to be appendicitis or cholecystitis during surgery, or considered to be malignant by pathologic results, and these, therefore, limited the comparability of the results. Besides, many other factors (such as patient age, gravidity, duration of pregnancy, weight gain, tocolytic treatment, mass size, the percentage of emergency operations undertaken, variation in the surgical procedures and the surgeon’s experience) may affect the clinical heterogeneity. Confounding bias cannot be ignored as the included studies were retrospective. There were no available data on pregnancy complications, nor was it possible to assess whether the effects of laparoscopic surgery on pregnancy outcomes were associated with other pregnancy complications. Therefore, further large-scale randomized trials are needed to confirm the present findings. However, it may be difficult to perform a randomized trial due to the particularity of pregnant women. In addition, the statistically significant difference in fetal outcomes was not possible to be identified owing to the limited number of studies available for pooling. Neither the allocation of surgical methods nor the assessment of outcome was blind, and it is important to bear in mind publication bias, particularly in meta-analytical research based on published studies.

## Conclusions

This study presented that laparoscopic surgery for pregnant women with adnexal mass was associated with less operative blood loss, reduced time in hospital and decreased rate of preterm labor. What is more, no significance was found in terms of fetal loss. More results should be awaited with particular interest on the outcomes mentioned above, given that only a few controlled trials published that limit exploration of the results to the clinical setting.
