# Correction to: Laparoscopy versus open surgery for adnexal masses in pregnancy: a meta-analytic review

**DOI:** 10.1007/s00404-019-05199-5

**Published:** 2019-07-11

**Authors:** Piaopiao Ye, Na Zhao, Jing Shu, Heping Shen, Yanpeng Wang, Lifeng Chen, Xiaojian Yan

**Affiliations:** 1 Department of Gynecology, The First Affiliated Hospital of Wenzhou Medical University, Wenzhou, 325000 China; 2 Department of Gynecology, Zhejiang Provincial People’s Hospital, Hangzhou, 310014 China

## Correction to: Archives of Gynecology and Obstetrics (2019) 299:625–634 10.1007/s00404-018-05039-y

The article listed above was initially published with incorrect copyright information. Upon publication of this Correction, the copyright of this article changed to “The Author(s)”.

The original article has been corrected.

